# *Crotalus Durissus Ruruima*: Current Knowledge on Natural History, Medical Importance, and Clinical Toxinology

**DOI:** 10.3389/fimmu.2021.659515

**Published:** 2021-06-08

**Authors:** Manuela B. Pucca, Paulo Sérgio Bernarde, Anderson Maciel Rocha, Patrik F. Viana, Raimundo Erasmo Souza Farias, Felipe A. Cerni, Isadora S. Oliveira, Isabela G. Ferreira, Eliseu A. Sandri, Jacqueline Sachett, Fan Hui Wen, Vanderson Sampaio, Andreas H. Laustsen, Marco A. Sartim, Wuelton M. Monteiro

**Affiliations:** ^1^ Medical School, Federal University of Roraima, Boa Vista, Brazil; ^2^ Laboratório de Herpetologia, Centro Multidisciplinar, Universidade Federal do Acre, Cruzeiro do Sul, Brazil; ^3^ Biology Department, Cathedral Faculty of Higher Education, Boa Vista, Brazil; ^4^ National Institute of Amazonian Research, Biodiversity Coordination, Laboratory of Animal Genetics, Manaus, Brazil; ^5^ Department of BioMolecular Sciences, School of Pharmaceutical Sciences of Ribeirão Preto, University of São Paulo, Ribeirão Preto, Brazil; ^6^ Insikiram Institute of Indigenous Higher Studies, Federal University of Roraima, Boa Vista, Brazil; ^7^ Department of Medicine and Nursing, School of Health Sciences, Amazonas State University, Manaus, Brazil; ^8^ Department of Teaching and Research, Alfredo da Matta Foundation, Manaus, Brazil; ^9^ Antivenom Production Section, Butantan Institute, São Paulo, Brazil; ^10^ Department of Teaching and Research, Dr. Heitor Vieira Dourado Tropical Medicine Foundation, Manaus, Brazil; ^11^ Department of Biotechnology and Biomedicine, Technical University of Denmark, Kongens Lyngby, Denmark; ^12^ Institute of Biological Sciences, Amazonas Federal University, Manaus, Brazil

**Keywords:** *Crotalus durissus*, *Crotalus durissus ruruima*, rattlesnake, snakebite, envenoming, venom, antivenom

## Abstract

*Crotalus durissus ruruima* is a rattlesnake subspecies mainly found in Roraima, the northernmost state of Brazil. Envenomings caused by this subspecies lead to severe clinical manifestations (e.g. respiratory muscle paralysis, rhabdomyolysis, and acute renal failure) that can lead to the victim’s death. In this review, we comprehensively describe *C. d. ruruima* biology and the challenges this subspecies poses for human health, including morphology, distribution, epidemiology, venom cocktail, clinical envenoming, and the current and future specific treatment of envenomings by this snake. Moreover, this review presents maps of the distribution of the snake subspecies and evidence that this species is responsible for some of the most severe envenomings in the country and causes the highest lethality rates. Finally, we also discuss the efficacy of the Brazilian horse-derived antivenoms to treat *C. d. ruruima* envenomings in Roraima state.

## *Crotalus Durissus Ruruima*: Natural History

**Figure 1:**
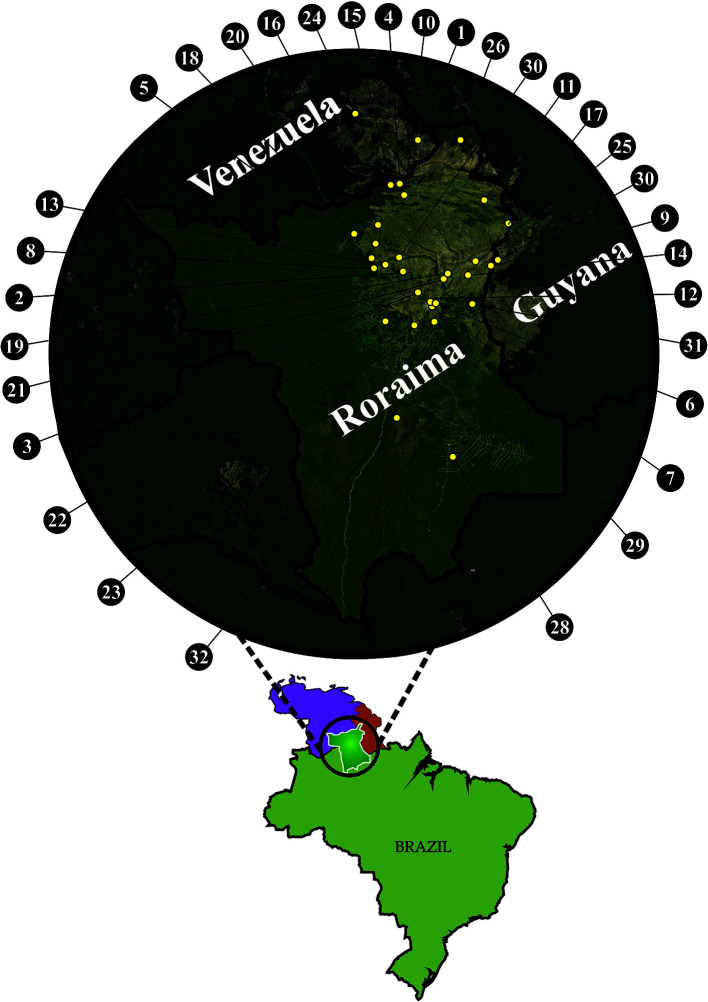
Map with records of *Crotalus durissus ruruima* in Brazil (BR), Venezuela (VE), and Guyana (GU): 1 - Paulo Camp., Mount Roraima, VE (5°00’N;60°52’W) (21); 2 - Taiano, Alto Alegre, RR, BR (3°15’N;61°04’W) (21); 3 - Bom Intento Farm, Boa Vista, RR, BR (2°58’N;60°52’W) (14); 4 -Pacaraima, RR, BR (4°25’N; 61°08’W) (21); 5 - Maracá Island, RR, BR (03°25’N; 61°29’W) (18); 6 - Boa Vista, RR, BR (02°49’N;60°39’W) (21); 7 - 7° BIS, Boa Vista, RR, BR (02°47’N; 60°41’W) (21); 8 - Maloca Mangueira, Alto Alegre, RR, BR (03°18’N;61°27’W) (21); 9 - Bonfim, RR, BR (03°21’N;59°49’W) (21); 10 - Surumu River, RR, BR (04°16’N;61°03’W) (21); 11 - Maloca Boqueirão, Alto Alegre, RR, BR (04°12’N; 59°59’W) (21); 12 - Monte Cristo Farm, Boa Vista, RR, BR (02°51’N; 60°42’W) (21); 13 - Salvamento Farm, Alto Alegre, RR, BR (03°20’N; 61°18’W) (21); 14 - Igarapé Garrafa, Boa Vista, RR, BR (03°12’N; 60°12’W) (21); 15 - Sorocaima, RR, BR (04°25’N; 61°11’W) (21); 16 - Três Corações, RR, BR (03°52’ N; 61° 24’ W) (this study); 17 - Normandia, RR, BR (03°51’N, 59°35’W) (14); 18 - Tepequém, RR, BR (03°45’N; 61°43’W) (21); 19 - Campo Alegre, Boa Vista, RR, BR (3°16’31.7”N 60°31’27.0”W) (14); 20 - Amajari, RR, BR (03°37’N; 61°26’W) (21); 21 - Passarão, Boa Vista, RR (03°11’N;60°35’W) (14); 22 - Apiaú, Alto Alegre, RR, BR (02°35’N;61°18’W) (14); 23 - Mucajaí, RR, BR (02°32’N;60°55’W) (14); 24 - Carimán-Paru, Gran Sabana, VE (0521N; 6142W) (4); 25 - Bonfim, RR, BR (03°23’N; 60°06’W) (14); 26 – Uiramutã, RR, BR (4°60’, 60.18’W) (21); 27 - Colônia Coronel Mota em Taiano, Alto Alegre, RR, BR (03 °26’N, 61.07’W) (21); 28 - Rorainópolis, RR, BR (0°46’27.7”N 60°24’13.6”W) (21); 29 - Vila Serra Grande, Cantá (this study), RR, BR (2°34’47’’N 60°38’57’’W); 30 – Lethem, GU (3°22’59’’N 59°48’17’’W) (14); 31 - São Francisco Village, Bonfim, RR, BR (2° 48’58’’N 60°08’34’’W) (this study); 32 - Viruá National Park – Caracaraí, RR, BR (1°17’39.3”N 61°09’04.6”W) (21). Georeferencing was made with QGis software and the final figure was prepared using CorelDraw.

The species *Crotalus durissus* is widely distributed in South America and occurs sporadically from Colombia to Argentina. It includes 11 subspecies (*C. d. durissus, C. d. cascavella, C. d. collilineatus, C. d. cumanensis, C d. marajoensis, C. d. maricelae, C. d. ruruima, C. d. terrificus C. d. trigonicus, C. d. unicolor and C. d. vegrandis*) (1). The highly uneven distribution of *C. durissus* in South America, which includes open habitats to the north and south of the Amazon rainforest as well as open relictual formations, adds considerable interest to phylogeographic studies of this complex species (1). In Brazil, this rattlesnake species (*C. durissus*) presents six subspecies (*C. d. durissus*, *C. d. cascavella, C. d. collilineatus, C. d. marajoensis*, *C. d. ruruima* and *C. d. terrificus*) (2). The subspecies *C. d. ruruima* was described by Hoge in 1966 (3), based on specimens collected at Mount Roraima in Venezuela (**Figure 1**). Among the six Brazilian rattlesnakes, the *C. d. ruruima* is one of the most intriguing subspecies due to its restricted distribution (limited to the northern area of the state of Roraima state in Brazil and southern Venezuela) and the unique biochemical and pharmacological properties of its venom (5, 6).

### Geographic Distribution

**Figure 2:**
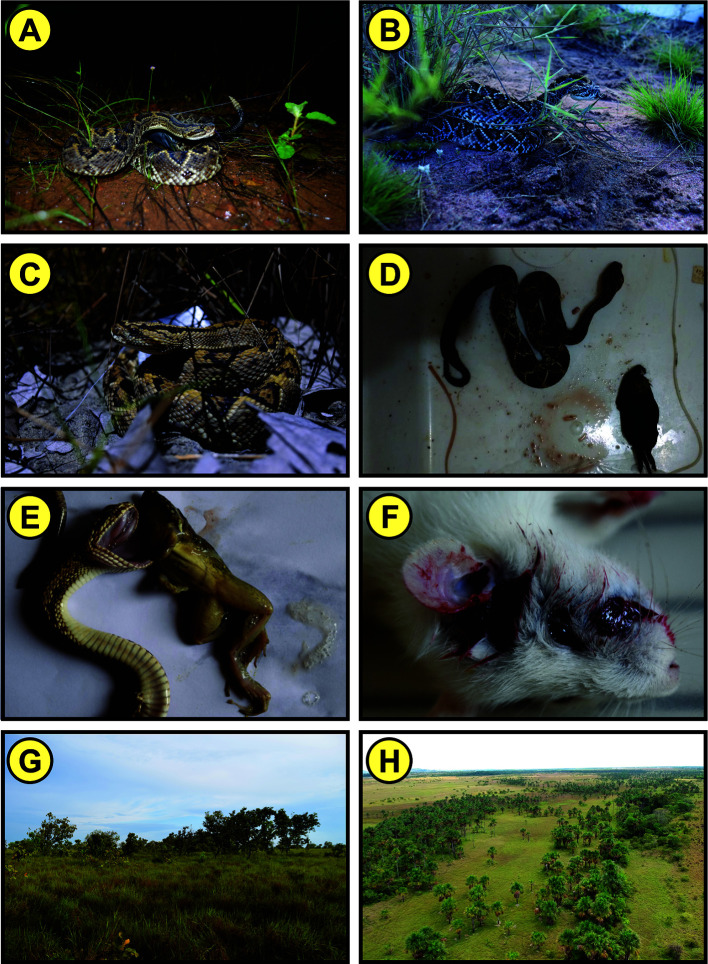
**(A–C)** *Crotalus durissus ruruima*; **(D)** Specimen of *C. d. ruruima* with rodent found in its stomach contents; **(E)** Amphibian *Leptodactylus macrosternum* regurgitated by a juvenile specimen of *C. d. ruruima*; **(F)** Mouse recently bitten by a captive *C. d. ruruima* showing massive bleeding; **(G)** Region of savanna (*lavrado*) where *C. d. ruruima* occurs; **(H)** Aerial image of the savanna (*lavrado*) area where *C. d. ruruima* occurs. Pictures: Anderson Maciel Rocha.

*C. d. ruruima* occurs in open areas of savannas (*lavrados*) in the state of Roraima (Brazil) and on the Mount Roraima slopes and Mount Cariman-Peru in the state of Bolívar (Venezuela) (7) (**Figures 2B, C, G, H**). It may also occur in adjacent territory of Guyana (7). In Roraima, *C. d. ruruima* has been recorded at the edge of forests near the municipality of Rorainópolis, in Viruá National Park in Caracaraí, in Caubi near the Mucajaí River, Apiaú, Taiano, Fazenda Salvamento, Maloca Boqueirão, Maloca da Mangueira in Alto Alegre, Vila Serra Grande-Cantá, Maracá Island, and to the west of Tepequém in Amajari. In the open regions (working land), it has been recorded close to Boa Vista city on the 7^th^ Infantry base, Bom Intento, Monte Cristo, Passarão, Campo Alegre, Igarapé Garrafa, and Igarapé Carrapato, and follows the open formations from Bonfim to Normandia and the far north of Pacaraima and Uiramutã.

*C. d. ruruima* is restricted to the open areas of the savannas, which is the expected behavior for this species whose life history is related to these plant formations (8). However, it has already been recorded in the Apiaú region probably by the intense deforestation that provided many open areas that the rattlesnake colonized. This type of population dispersion for *C. durissus* has already been recorded in Rio de Janeiro for subspecies *C. d. terrificus* (9).

### Taxonomy

*C. d. ruruima* was recognized by Peter & Orejas-Miranda (1970) (10) and Cunha & Nascimento (1980) (11) as a valid taxon. The geographically close subspecies, *C. d. dryinus* and *C. d. trigonicus*, were synonymized with *C. d. ruruima*. *C. d. trigonicus*, described by Harris & Simmons (1978) (12), was synonymized with *C. d. dryinus* by Abuys (1987) (13) and subsequently, following the recommendations of Cunha and Nascimento (1980) (11), with *C. d. ruruima* (14). Vanzolini & Caleffo (2002) (8) examined a paratype of *C. d. dryinus* and agreed with Rubio (1998) (14). Campbell and Lamar in 2004 (7) considered the validity of *C. d. trigonicus* questionable. Costa and Bérnils (2018) did not consider *C. d. trigonicus*, and indicated only *C. d. ruruima* for Roraima (2).

### Morphology

The name of the genus *Crotalus* derives from the Greek word “Krotalon”, which means “rattle” or “castanets” in reference to the characteristic appendix at the end of the tail of these snakes (7) (**Figure 2A**). The rattlesnake belongs to the Viperidae family and Crotalinae subfamily, which is characterized by presenting species with a loreal pit, vertical pupils and keeled dorsal scales (7). Similar to the other subspecies of *C. durissus*, *C. d. ruruima* has a distinct pair of dark stripes on the neck and a triangular head, which is distinguishable from the neck (7).

*C. d. ruruima* presents 167-170 ventral scales in males and 174-177 in females, and 25-29 subcaudal scales in males and 21-23 in females (3). In relation to the coloring pattern, the paravertebral bands are distinctly marked with white and lighter scales in the center, with the same light center pattern and white outer edges in the head markings (3). The dorsal diamonds are less contrasted than in *C. d. durissus* and sharply surrounded with white, thus approaching the pattern of the specimens of *C. d. terrificus* from the southern end of Brazil. Some specimens have a tendency to have the obliterated pattern of *C. d. vegrandis*.

### Size

The total body length of *C. d. ruruima* varies between 315 mm and 1345 mm, and the average length is 839 mm (15). The smallest male and female presented 368 mm and 315 mm, respectively, and the largest male and female presented 1250.9 mm and 1115 mm, respectively (15). Female specimens tend to be slightly larger, presenting a mean rostro-cloacal length of 630.9 mm (n=22), while males presented 627.3 mm (n=41) (15). However, sexual dimorphism was not statistically evident in *C. d. ruruima*; thus the analysis of a greater number of specimens is necessary for a better understanding of possible differences in body length (15). Generally, in species in which male individuals are larger than female, male-to-female combat occurs (16), and this behavior has already been witnessed in populations of *C. durissus* in southeastern Brazil (17, 18).

### Habitat and Activity

Rattlesnakes of the species *C. durissus* are characteristic of open areas (savannas), while it can also occur in areas that have been altered by human activity (pastures and crops) and forest edges (9, 15, 19–23). In Roraima, this snake is present in the open areas of savannas, woods edges, areas modified by the crop plantings (e.g. corn and soybean), and acacia (*Acacia* sp.) plantations and pastures (15, 19, 22).

The rattlesnakes present greater activity during the night twilight period, and are relatively inactive at the beginning of the day (6 to 8 am), at this time usually going from areas such as firebreaks to their nests (15). During the afternoon, they usually do not present activity; thus, a large part of the specimens are observed in this period inside their burrows (15). During the twilight-night period, these rattlesnakes present greater activity between 7 pm and midnight, with their activity peak between 9 pm and 10 pm (15).

When they are not hunting during the day, they remain inside their burrows and among grasses and shrubs (15), probably because these microhabitats favor thermoregulation and also contribute to the protection from possible predators (20). In this situation, where the snake is partially covered by vegetation, its visual detection becomes more difficult (24). Thermoregulation constitutes the main diurnal activity of nocturnal Viperidae snakes (20, 24), which may explain most cases of snakebite in Roraima state, since this period is also that of human activity in the area (25, 26).

### Diet and Foraging Activity

*C. durissus* is terrestrial and hunts mainly by waiting for rodents (20, 21, 27) (**Figure 2F**). Small mammals, mainly rodents, make up most of the diet of *C. durissus* in the southeastern (27, 28) and central regions (29) of Brazil, though lizards (*Ameiva ameiva*) are also consumed in a smaller proportion. Birds have also been recorded in the diet of northeastern populations (30) (**Figure 2D**). Indeed, rodents were the main findings in the stomach contents of *C. d. ruruima* and, less frequently, lizards (*Tropidurus hispidus*) (15). Unlike other populations of *C. durissus*, anurans (*Leptodactylus macrosternum*) were found in *C. d. ruruima* stomachs (31) (**Figure 2E**), which may be related to differences observed by some authors (6) in the venom of this subspecies.

In relation to the hunting strategy, just as in the populations of other subspecies (20, 23), *C. d. ruruima* hunts by waiting on the ground, seeking preys that exhibit surface activity (rodents, lizards e.g., *T. hispidus* and the amphibians e.g., *L. macrosternum*) (15). In the burrows where they usually shelter, there have been cases in which 5 or 7 rattlesnakes were found occupying the same burrow (15).

### Seasonality

During the year, *C. d. ruruima* shows greater activity from August to February (15), coinciding with warmer and drier days, which have an average temperature of 27 °C and average humidity of 71.2%.

### Reproduction

*C. durissus* is a viviparous species, giving birth to an average of 11 young in southeastern Brazil and between 12 and 33 in northeastern Brazil (32). The populations of the southeast present long vitellogenesis, beginning in March, with gestation between October and January and the birth of the offspring between January and March (33, 34). Male individuals can perform combat ritual (17, 18).

There is little information regarding aspects of reproduction of *C. d. ruruima* (15). Males are more active throughout the year with peaks in the months of August to November, while females are more frequent in the months of December to the end of January (15). Juveniles are more frequent during the months of March to May (15), during the rainy period in Roraima (March to June) (35), which probably corresponds to the birth period in offspring. Two pregnant females were recorded during the month of January, one has the total body length of 580 nm with seven embryos and the other of 730 mm with nine.

### Defensive Behavior

When a human approaches, the rattlesnake (*C. durissus*) most often flees but may turn the head and anterior region of the body in the direction of the observer, while shaking the rattle of the tail and curling itself up (21). Usually, this snake only shakes its rattle when a person comes very close to it (about a meter or less) (21). Sawaya et al. also studied the defensive behavior of *C. durissus* in the Cerrado area of southeastern Brazil. When handled, it was observed that this snake can shake the rattle of the tail, strike a bite, perform cloacal discharge, open its mouth, struggle, bite, squirt liquid from the cloacal gland in the form of jets, and flatten and rotate the body. The secretion from the cloacal gland of *C. durissus* has a strong smell and can cause nausea and burning in the mouth and eyes, indicating that this can be an important defensive tactic against predators (21).

### Genetics

Several molecular analyses, which included different lineages of *C. durissus*, have generated strong support for monophyly. This includes the lineages of *C. durissus*, *C. simus*, *C. tzabcan* (southern lineages), and *C. culminatus*, together with two endemic species recently described for Central America: *C. mictlantecuhtli* and *C. ehecatl* (northern lineages) (1, 36–40).

All subspecies of *C. durissus* that occur in Brazil are sister groups to *C. d. vergrandis* and *C. d. cumanensis* from Venezuela (40). However, *C. d. ruruima*, together with *C. d. durissus* from Guyana (previously assigned as *C. d. dryinas*) form an arrangement clearly separated from the other subspecies found in Brazil. Furthermore, comparative genomic hybridization involving ancient and advanced lineages of snakes has shown that *C. d. ruruima* and *C. d. terrificus* (Brazilian subspecies) possess different landscapes of repetitive sequences in their genomes, that are likely associated with particular differentiation processes at species level (41). This highlights that the real diversity of the Neotropical rattlesnake complex is currently underestimated and it is yet to be fully investigated. As such, this would require comparative analyses encompassing all extant subspecies of South American *C. durissus* lineages.

## *Crotalus Durissus Ruruima:* Venom Composition

Snake venomics enables the qualitative and quantitative understanding of venoms of different species (42). There are few studies comparing the venomics of Brazilian rattlesnake subspecies (5, 43, 44). Interestingly, while originated from the same species (*i.e. C. durissus*), the Brazilian rattlesnake subspecies present significant differences in venom composition (**Table 1**). Venom of *C. d. ruruima* presents a great variability, with repercussions even in the color, what made the first researchers of this subspecies’venom to classify it in “white” and “yellow” venom (49), which was also observed in *C. d. terrificus* and *C. viridis helleri* venoms (50, 51). The yellow color of venoms is closely related to the presence of L-amino acid oxidases (LAAOs) (52), which will be discussed later. In this section, the main identified and/or isolated protein classes from *C. d. ruruima* venom are explored.

**Table 1:** Venomic comparison of Brazilian *Crotalus durissus* subspecies.

C. durissus	Crotoxin	SVSP	CTL	SVMP				Crotamine	LAAO	BIP	Others	Ref.
				I	II	III	IV					
*ruruima*	82.7	8.1	4.3	–	–	2.9	–	1.5	<0.5	<0.1	–	(5)
*cascavella*	72.5	1.2	<0.1	–	–	<0.1	–	–	<0.1	–	20.3	(44)
*collilineatus*	67.4	1.9	<0.1	–	–	0.4	–	20.8	0.5	–	13.8	(44)
*durissus*	NR											
*marajoensis*	NR											
*terrificus*	48.5-82.7	0.7-25.3	<0.1-2.7	0.09-5.5				1-19	0.6-4.5	1.8	0.5-22.3	(5, 45–48)

BIP, bradykinin-inhibitory peptide; CTL, C-type lectin-like; LAAO, L-amino acid oxidase; NR, not reported; SVMP, snake venom metalloprotease; SVSP, snake venom serine protease.

### Crotoxin (CTX)

One of the most abundant protein family in *C. durissus* snake venoms is the CTX family (44, 45, 53). Crotoxin is a heterodimer protein composed of a complex of a basic subunit named phospholipase A_2_ (PLA_2_) and a non-toxic acidic subunit, crotapotin, which prevents the PLA_2_ binding in to non-specific sites (54–56). Among subspecies CTXs’ abundance varies from 48.5 to 82.7% of total venom composition, found more abundant in *C. d. ruruima* venom (**Table 1**) (5). Crotoxin-rich venoms from Neotropical *Crotalus* subspecies are classified into type II phenotype, characterized by a high lethality (57). CTX is responsible for neurotoxicity and myotoxicity, both important events on pathophysiology (5, 58–60). Studies have also brought novel perspectives for CTX as a possible pharmacological strategy due to its anti-inflammatory/immunosuppressive, anti-tumoral, and microbicidal effects (61–63).

### Phospholipases A_2_ (PLA_2_s)

Snake venom PLA_2_s constitute a puzzling group of molecules since, despite having a similar three-dimensional structure and highly conserved molecular regions, display a plethora of pharmacological activities such as myotoxic, neurotoxic, anticoagulant, hypotensive, hemolytic, platelet aggregation inhibition, bactericidal, and pro-inflammatory activities (64).

Secretory PLA_2_s occur in a large variety of venoms (*e.g.* snakes, arthropods, and mollusks), being able to cleave the *sn*-2 acyl bond of glycerophospholipids, releasing free fatty acids and lyso-phospholipids (65). Several studies have shown that *C. d. ruruima* venoms contains various isoforms of PLA_2_ with distinct functions (6, 66–68). Cdr-12 and Cdr-13 are two isoforms that were isolated from *C. d. ruruima* snake venom. They present high content of Lys, Tyr, Gly, Arg, and 14 half-Cys residues, typical of a basic PLA_2_ and show high homology to other D49 PLA_2_s isolated from venoms of crotalic snakes (66).

Also, the crotoxin-like toxins were capable to induce myonecrosis and edema in mice, and a potent blockade of neuromuscular transmission in chicken biventer cervicis preparation (66). Cavalcante and colleagues (2015) also observed that a crotoxin isolated from *C. d. ruruima* “white” venom was capable to induce a neuromuscular blockade of indirectly evoked twitches of mice phrenic-diaphragm preparations (68).

Another PLA_2_ isolated from *C. d. ruruima* venom is PLA_2_A, a calcium dependent enzyme which shows antibacterial activity (67). The most recent PLA_2_ isolated from *C. d. ruruima* is CBr (basic crotoxin), which presents its 20 amino-terminal residues identical to CB1 from *C. d. terrificus* venom. The CBr along with the whole venom were able to activate macrophages with focus on the formation of lipids droplets and synthesis of lipid mediators, suggesting its role on the production of inflammatory mediators during envenomings (6). Moreover, these PLA_2_s could be responsible to potentiate toxicity of venoms, according to the synergism phenomenon (69).

### Snake Venom Serine Proteases (SVSPs)

SVSPs comprise a group of extensively studied enzymes, widely found in the venom of terrestrial snakes from Viperidae, Elapidae, and Crotalidae families (70). SVSPs are catalytically active proteins able to degrade fibrinogen (71), resulting in several biological effects such as hemorrhagic, procoagulant, anticoagulant, platelet activation, and bradykinin-release (72).

Based on their functions, the procoagulant snake venom proteases are classified as factors I (snake venom thrombin-like enzymes, SVTLEs), V, VII and X, and groups C and D prothrombin activators (73). SVTLEs are the prevalent class of SVSPs from Viperidae venoms and present similar activities to human thrombin (74, 75). The SVTLEs are considered multifunctional toxins due to their broad substrate specificity and can act on different biological systems of the preys or the victims. Therefore, the investigation of the intrinsic pathways involved in the variety of biological activities of these molecules may contribute to expanding their potential applications (76).

Although *C. d. ruruima* venom presents SVSPs (8.1%) (5), these enzyme classes have never been isolated from this venom, unlike from *C. d. terrificus* (0.7-25.3%; **Table 1**) (5, 45–48), from which two SVSPs have been isolated, gyroxin and CdtSP2, presenting important roles in coagulation disturbances, neurotoxicity, and inflammation (77–79). Studies have also shown potential therapeutic use of SVSPs from *C. durissus* subspecies, such as the use for fibrin sealant in the treatment of dermatological ulcers (80). Also, an SVSP from *C. d. collilineatus* venom (1,9%) (44, 81) was recently shown to modulate ion channels (hEAG1, Kv10.1, KCNH1), helping to understand and reveal the complex and pluripotent pharmacology of SVTLEs, and also to open perspectives in terms of applicability particularly in the field of oncology due to its action on the oncogenic *ether-a-go-go* 1 voltage-gated potassium channel (70).

### C-Type Lectin-Like Proteins (CTLs)

*C. d. ruruima* is the Brazilian subspecies that presents the highest abundance of CTLs (4.3%) (5), while the others subspecies present lower proportions in their venoms (<2.7%) (5, 45–48). Snake venoms CTLs are heterodimeric proteins, composed of two homologous subunits α and β (with 14-15 kDa and 13-14 kDa, respectively), which can be arranged in oligomeric complexes (82, 83). They show low primary sequence similarities to venom sugar-binding lectins, with no ability to interact with glycans. However, CTLs show an indiscriminate ligand spectrum, targeting clotting factors and several receptors on platelets, endothelial cells, and immune cells. This results in a wide range of pharmacological activities, acting in hemostasis and inflammation (84). There are no studies that disclose the isolation and characterization of this protein class from *C. d. ruruima*. Nonetheless, others CTLs have been described in *C. durissus* venoms. Convulxin, isolated from *C. d. terrificus* consists of the most studied CTLs, acting as a platelet-aggregating agonist that acts on the p62/GPVI collagen receptor in platelet surface (85). Other CTL is crotacetin, a convulxin-homologue toxin isolated from *C. d. terrificus*, *C. d. cascavella*, and *C. d. collilineatus* venoms with platelet aggregating and antibacterial activity (86).

### Snake Venom Metalloproteases (SVMPs)

SVMPs comprise a group of metal-dependent proteases, playing a critical role in the proteolytic and biological activities of the venom (87). Zinc-dependent SVMPs belong to the metzincin family, which has a zinc-binding domain in common and structures very similar to each other. The zinc-binding site of this family has the amino acid sequence that is common to all subfamilies (88).

Metalloproteases are classified into three classes (PI, PII, and PIII) according to the organization of their multi-domains, considering the presence or absence of non-proteolytic domains observed in mRNA transcripts and in isolated proteins from snake venom (89). The PI-SVMPs are composed only of a metalloprotease domain. The PII-SVMPs have a metalloprotease domain followed by a disintegrin domain, which are often separated by a post-translational proteolytic cleavage. Both proteolytic products are stable (89). Finally, the PIII-SVMPs have a cysteine-rich domain (Cys-rich), in addition to metalloprotease and disintegrin-like (dis-like) domains. The PIII is subdivided into subclasses (PIIIa, PIIIb, PIIIc, and PIIId), reflecting the potential proteolytic processing and formation of dimeric structures, for which PIII has an additional lectin-like domain (89, 90).

According to the venomic approach, *C. d. ruruima* presents only PIII-SVMP in its venom composition (2.9%) (5), as well as *C. d. cascavella* (<0.1%) and *C. d. collilineatus* (0.4%) (44), although the latter also presents disintegrins (44, 53), while *C. d. terrificus* presents varied proportions of the SVMPs group (0.09-5.5%; **Table 1**) (5, 45–48). Although SVMPs from *C. durissus* subspecies are poorly studied, this group of toxins from *Crotalus* spp. from North and Central Americas are more abundant and have been widely investigated. These studies have shown hemostatic effects such as fibrino(geno)lytic and inhibition of platelet aggregation activities as well as local hemorrhage caused by degradation of the capillary basement membrane and muscle damage with lower regeneration (91–93). As *C. d. ruruima* venom composition differs from other more prevalent *C. durissus* subspecies envenomings, an antivenom based on oligoclonal mixtures of antibodies could be a strategy to improve treatment.

### Crotamine

Crotamine, which may or may not be present, is one of the main components of rattlesnake venoms. This particularity indicates a Mendelian character of the toxin, since its frequency increases according to the east-west and north-south axes in Brazilian territory (44, 94). Although *C. d. ruruima* venom can present crotamine in their venom cocktail, its abundance is very low (1.5%) as shown in preliminary results (5) when compared to *C. d. collilineatus* venom (20.8%) (44). Crotamine has an evident myotoxic action during envenomings, which was demonstrated through increased levels of creatine phosphokinase (CK) *in vivo* (53, 58, 95), as well as neuromuscular blocking effects (96) and hemostasis modulation (79, 97).

### Bradykinin-Inhibitory Peptide (BIP)

*C. d. ruruima* and *C. d. terrificus* are the only Brazilian rattlesnakes presenting BIP, even in a low proportion (<1.8%) (5). This molecule was found in other Crotalinae venoms (e.g. *C. viridis viridis*, *Lachesis muta*, and *Agkistrodon bilineatus* venoms), which present a fully conserved primary structure, suggesting a conserved biological role for this toxin (98). BIPs present antagonistic effects on the vasodilatation induced by bradykinin at bradykinin receptor type 2 (B2 receptors), which are expressed in vascular endothelial and smooth muscle cells (98, 99), resulting in vasoconstriction effects by disrupting the functioning of the cardiovascular system, thus also contributing to clinical effects of envenomings (98).

### L-Amino Acid Oxidases From Snake Venoms (SV-LAAOs)

SV-LAAOs are widely distributed in venomous snake families of Viperidae, Crotalidae, and Elapidae (52). LAAOs are flavoenzymes that catalyze the stereospecific oxidative deamination of an L-amino acid substrate, producing α-keto acid, ammonia, and hydrogen peroxide (H_2_O_2_) (100–102). With a molecular mass of around 110-150 kDa under non-denaturating conditions, SV-LAAOs are homodimeric glycoproteins linked to flavin adenine dinucleotide (FAD) (103, 104). As a prosthetic group of LAAOs, FAD has riboflavin, which characterizes the yellow color of venoms (52).

An interesting aspect of *C. d. ruruima* venom concerns its color, presenting a “white” and “yellow” variation with particular biological activities (49). The proteome analysis performed by Calvete and colleagues from *C. d. ruruima* was performed with a pool of “white” venom, and showed a low abundance of LAAO (<1%) (5). In other, *C. durissus* subspecies venoms, LAAOs are also present in low abundance (44), with a highest concentration of 4.5% found in a *C. d. terrificus* sample (**Table 1**) (5, 45–48). LAAO’s physiological role is still unknown; it is speculated that they may be related to venom conservation and stabilization of the venom glands, due to their antibacterial properties (102). LAAOs have been isolated from *C. durissus* venoms, showing hemolytic activity, induced plasma clot and platelet aggregation (105–107). Many of these could be related, at least in part, with H_2_O_2_ produced during the chemical reaction catalyzed by LAAOs, contributing to envenoming toxicity, due to oxidative stress (108, 109).

### *C. d. Ruruima* Specific Features

Although the main rattlesnake toxins were described in *C. d. ruruima* venom using proteomics (5), many toxins encountered in other Brazilian *C. durissus* subspecies (e.g. disintegrins, hyaluronidases, growth factors, nucleases, and nucleotidases), which are also determinants for the toxicity of venoms, have not been described for this subspecies (5, 43–46, 53, 110, 111). However, we do not exclude the possibility of *C. d. ruruima* presenting other compounds. Dos-Santos and colleagues evidenced individual venom variations between six *C. d. ruruima* snakes: 1) the toxicity of white venoms was higher than yellow venoms; 2) PLA_2_ activity also varied, being higher in yellow venom; 3) only one white venom showed hemorrhagic activity; 4) myotoxicity degree and edematogenic activity varied between venoms; and 5) coagulant activity in human plasma or bovine fibrinogen also varied between tested venoms (112). Another study with white and yellow venoms from *C. d. ruruima* demonstrated that they have similar lethality and coagulant activity, while presenting small differences regarding proteolytic, hemorrhagic, and necrotic activities (49). Cavalcante and colleagues (2015) also observed that *C. d. ruruima* “white” venom was capable of inducing a neuromuscular blockade of indirectly evoked twitches of mice phrenic-diaphragm preparations (68).

*C. d. ruruima* venom is the one with the highest abundance of crotoxin. Based on the high proportion of crotoxin, *C. d. ruruima* venom could be considered the most toxic Brazilian rattlesnake venom, since its “lethal neurotoxicity coefficient” (LNC) is higher than the other Brazilian rattlesnakes already studied (5, 44). This coefficient is determined through the ratio of average venom LD_50_ and the neurotoxins concentration (crotoxin and crotamine) (5). This fact could justify the severe cases of envenoming and lethality caused by *C. d. ruruima* snakes (26, 59).

## Human Envenomations Caused by *C. d. Ruruima*

### Epidemiological Aspects

In Brazil, snakebites are compulsorily recorded by the *Sistema de Informação de Agravos de Notificação* (SINAN, Brazilian Notifiable Diseases Surveillance System), based on data report forms used in the investigation and follow-up of cases of envenomings caused by animals. In the state of Roraima, in the extreme north of Brazil, *C. d. ruruima* is known to be the unique subspecies of *C. durissus* responsible for the local envenomings. Thus, the description of crotalid envenomings registered in SINAN in this state can provide important information about the epidemiological and clinical features of envenomings by *C. d. ruruima*. Of a total of 3,616 snakebites (71.3 cases/100,000 persons/year) reported to SINAN from January 1, 2010, to December 31, 2019, in Roraima, 396 (10.9%) cases were caused by rattlesnakes, resulting in an incidence rate of 7.8 cases/100,000 people/year.

**Figure 3:**
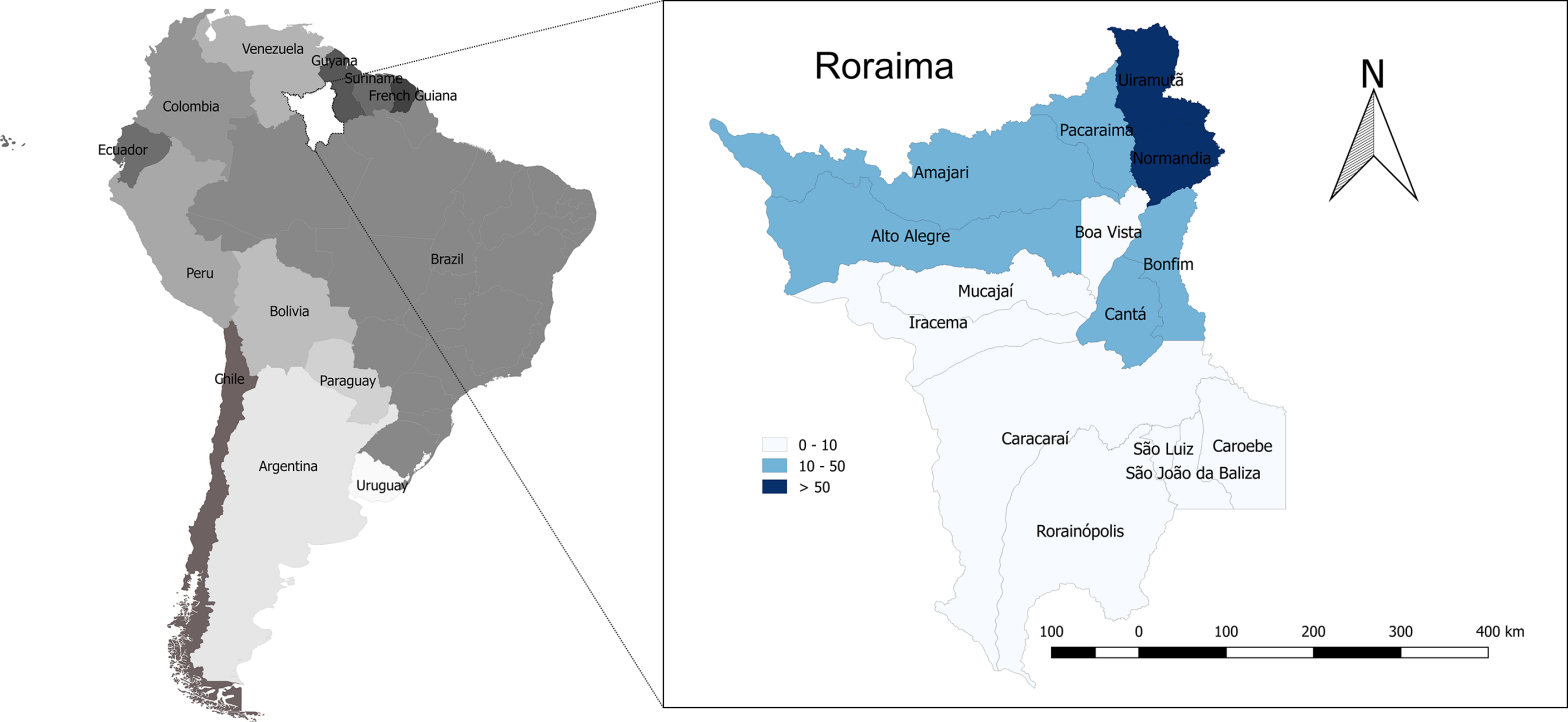
Geographic and temporal distribution of *Crotalus durissus ruruima* snakebites in the state of Roraima, Brazilian Amazon. Incidence rate per municipality (cases/100,000 inhabitants/year, showing a higher incidence in the north of the state).

Spatial distribution of rattlesnake bites across the Brazilian Amazon showed higher incidence in areas of savanna in high altitude areas (113). In Roraima, the geographic distribution of *Crotalus* envenoming shows the highest incidence rate in the northernmost municipalities, in the border with the Guyana and Venezuela, namely in the municipalities of Uiramutã and Normandia (**Figure 3**), coinciding with savannas that occur in mountainous areas in the northern areas of the state. There was a slight variation in the annual incidence rates during the study period. Incidence was higher in 2018 (94 cases; 16.3 per 100,000 inhabitants) and 2017 (54 cases; 10.3 per 100,000) and lower in 2010 (11 cases; 2.4 per 100,000). The seasonality of the cases was modest, with a slight increase in cases from the beginning of the rainy season.

Most of the snakebites occurred in males (77.3%). The most affected age group was between 16 and 45 years old (51.0%). Strikingly, Amerindians represent 11% of the Roraima’s total population but suffered 64.9% of reported *Crotalus* envenomings; this incidence is ~12 times higher than in the non-Amerindian population. In the Amazon, the distribution of the snakebite burden is disproportional among indigenous and non-Amerindian populations. In the state of Amazonas, the prevalence is 7.5 times higher among Amerindians (114). Indigenous people’s way of life, and their daily subsistence activities, involve daily interaction with snakes, without personal protective equipment, thus increasing the risk of snakebites (114). Regarding the area of occurrence, 93.0% were reported in rural areas; 24.3% of the snakebites were related to work activities. Regarding time elapsed from the bite until medical assistance, 57.0% of the cases received treatment within the first six hours after the snakebite, 22.7% within 6–24 hours, and 20.3% with more than 24 hours after the bite. Among the victims, 14.6% were illiterate, and 36.1% had ≤4 years of study. Most of the snakebites occurred in the lower (85.6%) and upper limbs (13.1%) (**Table 2**). This epidemiological profile confirms small case series previously published in the state of Roraima (25, 26, 115). Asato and colleagues showed that the *C. ruruima* envenomings attended to in Boa Vista, the capital of the state of Roraima, predominantly involved farmers during morning or afternoon work in their fields (26).

**Table 2:** Epidemiological characteristics of *C. d. ruruima* envenomings reported in the state of Roraima, northern Brazilian Amazon, from 2010 to 2019.

Variable (Completeness)	Number	%
**Sex (n=396; 100%)**		
Male	306	77.3
**Age range, in years (n=396; 100%)**		
0-15	113	28.5
16-45	202	51.0
46-60	51	12.9
≥61	30	7.6
**Ethnicity (n=379; 95.7%)**		
White	10	2.6
Black	13	3.4
Asian	2	0.5
Mixed	108	28.4
Amerindian	246	64.9
**Area of occurrence (n=384; 97%)**		
Rural	357	93.0
Urban	27	7.0
**Work-related (n=296; 74.7%)**		
Yes	72	24.3
**Education level, years (n=219; 55.3%)**		
Illiterate	32	14.6
≤4	79	36.1
5-8	49	22.4
>8	59	26.9
**Site of the bite (n=388; 98%)**		
Head	4	1.0
Trunk	1	0.3
Lower limbs	332	85.6
Upper limbs	51	13.1
**Time from bite to medical assistance, hours (n=375; 94.7%)**		
≤6	214	57.0
>6-24	85	22.7
>24	76	20.3

### Clinical Aspects and Physiopathology

**Figure 4:**
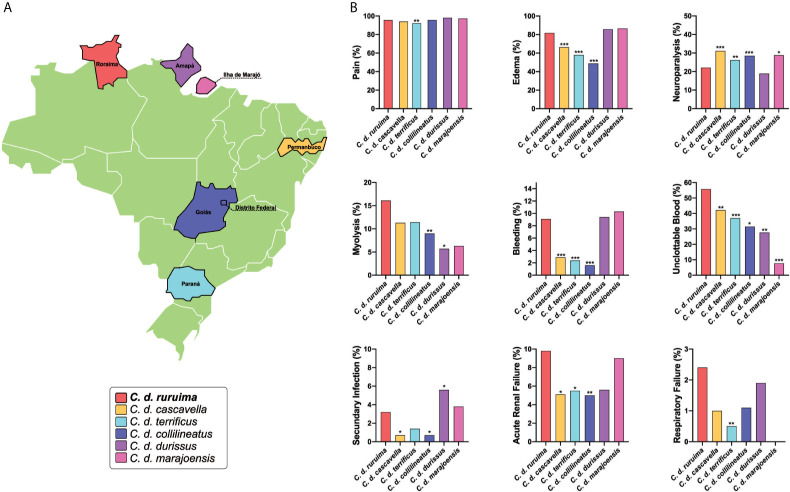
Cross-country comparison of local and systemic manifestations in envenomings caused by different *Crotalus durissus* subspecies. Data was gathered from the SINAN, from 2010 to 2019. Information was stratified by state, considering the geographic distribution of six *C. durissus* subspecies. **(A)** Only states without subspecies overlap in geographic distribution were selected. For statistical comparison, X-square or Fisher’s tests were performed in STATA software (StataCorp. 2013: Release 13. College Station, TX, USA), using the group of patients envenomed by *C. d. ruruima* as the reference. After the univariable analysis, any difference in the frequency of clinical manifestations between *C. d. ruruima* and another subspecies envenoming at a significance level of *p*<0.20 were included in a multivariable analysis, adjusting by age, sex, and time to medical assistance. Statistical significance was considered if *p*<0.05 in the Hosmer-Lemeshow goodness-of-fit test. **(B)** Only clinical manifestations with any significant difference are presented. Number of patients according *C. durissus* subspecies: *C. d. ruruima* (n=396), *C. d. cascavella* (n=990), *C. d. terrificus* (n=1,016), *C. d. collilineatus* (n=2,093), *C. d. durissus* (n=54), *C. d. marajoensis* (n=80); *p < 0.1; **p < 0.05; ***p < 0.01.

There is very little information in the literature on the clinical manifestations of envenomings caused by *C. d. ruruima*. However, the few reports suggest some differences in relation to the clinical pictures resulting from different subspecies of *C. durissus* found in South America. The main difference pointed out is the high frequency of inflammatory manifestations at the bite site and coagulopathy (26, 115). However, the small number of published cases prevents a more accurate comparison with data from other regions. To explore this review’s mentioned hypothesis and to present a more accurate comparison of envenomings caused by different subspecies of *C. durissus*, we analyzed the data gathered by SINAN, from 2010 to 2019, comparing envenomings cases of *Crotalus* occurring in six regions of the country in which the snake is present (**Figure 4**). Only country units (states) without subspecies overlap in geographical distribution of six *C. durissus* subspecies, according to the current knowledge (1, 2), were selected for comparison, using the group of patients envenomed by *C. d. ruruima* (cases from the state of Roraima) as the reference. As the epidemiological characteristics of the cases are different in relation to demographics and access to the health system, the results were statistically adjusted by sex, age, and time until the assistance. Results must be interpreted with caution, since a limitation of this approach is related to the lack of standardization in the clinical management of cases and data collection in different country regions, and the number of cases described for each subspecies differs widely. However, we believe that the broad population coverage allowed the acquisition of valuable information to guide future studies.

#### Bite Site Manifestations

In general, the authors report that local manifestations are usually discreet during envenomings caused by *C. durissus*, as a result of the low inflammatory activity of the venom (116–118). The most frequent local manifestation among *Crotalus* envenomings reported to SINAN was pain, ranging from 92.1% in *C. d. terrificus* to 98% in *C. d. durissus* (**Figure 4**). In *C. d. ruruima* envenomings, frequency of pain (95.6%) was significantly higher only than *C. d. terrificus* (**Figure 4**). Some studies have shown that pain may be absent in a large proportion of patients in São Paulo, where *C. d. terrificus* is the snake mainly responsible for envenomings (117). Edema (81.8%) was also very common in *C. d. ruruima* cases, at a higher frequency than observed for *C. d. cascavella* (66.4%), *C. d. terrificus* (58%), and *C. d. collilineatus* (48.9%) (**Figure 4**). Unfortunately, paresthesia is not reported by SINAN but is also a very common manifestation in *Crotalus* bites. In *C. d. ruruima* envenomings, paresthesia frequency ranges from 12.5 to 16.2% (26, 115). In the state of São Paulo, paresthesia was reported in 47.6% of the children (117) and 26.1% (116) of the general cases of *Crotalus* bites.

Local complications such as secondary infections and compartment syndrome seem to appear at very low frequency in *Crotalus* bites (119, 120). Data from SINAN show that secondary infections frequency ranged from 0.7% in *C. d. cascavella* and *C. d. collilineatus*, to 5.6% in *C. d. durissus*. In *C. d. ruruima* envenomings, the frequency of secondary infections (3.2%) was significantly higher than *C. d. cascavella* and *C. d. collilineatus*, but lower than *C. d. durissus* (**Figure 4**). Necrosis, compartment syndrome, functional loss and amputation were rare, and their frequencies were similar between envenomings caused by different *C. durissus* subspecies.

Local manifestations are commonly associated with the inflammatory properties that venom can trigger once inoculated. Although toxins from *C. durissus* subspecies are known to present low immunogenicity, studies have shown the capacity of venoms and toxins to promote local and systemic inflammatory responses in animal models (78, 121, 122). As previously described, *C.d. ruruima* “yellow” and “white” venom variations are responsible for inducing mouse paw edema (112). Considering that the venom components crotoxin and serine proteases are found to trigger an inflammatory response (78, 122), the higher frequency in local manifestations, such as edema, found in Roraima state could be associated with *C. d. ruruima* venom composition, in which the two toxins classes comprise over 90%. Moreover, although the low abundance of SVMP found in *C. d. ruruima* venom can be associated with rare cases of local sequelae, this group of toxins could be an additional factor for local injury as observed for North American *Crotalus* spp. venoms (93).

#### Rhabdomyolysis and Acute Renal Failure

The myotoxic activity is a result of the venom-induced skeletal muscle breakdown, often associated with rhabdomyolysis (123). In the state of Roraima, dark urine (13.5%) and myalgia (~25%) were reported after *C. d. ruruima* envenomings (26, 115). In the state of São Paulo, myalgia and dark urine were detected in 38.6 and 36.1% of the patients, respectively (118). In Minas Gerais, myalgia was detected in 29% and dark urine in 40% of the patients (124). In children from the same region, frequencies of myalgia and dark urine were still higher, exceeding 70% of the patients (116, 117). In the analysis of the SINAN database, rhabdomyolysis was significantly higher in patients bitten by *C. d. ruruima* (16.1%) using univariable analysis, compared to all other subspecies. However, after adjusting by sex, age, and time to medical assistance, significant difference remains only for *C. d. collilineatus* (**Figure 4**). The non-standardized collection of these signs and symptoms may prevent accurate comparisons across studies. In *C. d. terrificus*, serum CK activity increases early, reaching a peak in the first 24 hours after the bite, remaining above reference levels for more than four days in some patients (116, 117). In envenomings caused by *C. d. terrificus* (117, 118, 124), *C. d. collilineatus* (124, 125), *C. d. marajoensis* (126), and *C. d. ruruima* (26), CK activity was above normal levels in ~90% of the patients (118, 124, 125). Serum activities of lactate dehydrogenase and liver transaminases are also elevated in most cases. Hypocalcemia may occur in patients with intense rhabdomyolysis after *C. d. terrificus* bites (116). Both crotoxin and crotamine are the major myotoxic components within *C. durissus* venoms (96, 127). Moreover, crotoxin exhibits a capacity to spread to distant muscles and induce rhabdomyolysis, due to its systemic distribution ability mediated by the heterodimeric complex formation of the toxin to bind to specific cell targets (128). *C. d. ruruima* crude venom showed myotoxic activity, as observed for increased plasma levels of CK and histopathological analysis of myonecrosis (49, 112). Isolated crotoxin from this venom also induced increased plasma levels of CK and LDH not only at intramuscular administration but also intraperitonealy, confirming the systemic effect of CTX (66). Considering the role of crotoxin in muscular damage events and its high abundance in *C. d. ruruima* venom, the high frequency of myonecrosis in patients from Roraima state could be associated with CTX activity.

As a consequence of rhabdomyolysis, a major complication following *Crotalus* snakebites is acute renal injury (123). The definitions of acute renal failure vary widely across studies, and this lack of standardization is also a bottleneck for comparisons. In *C. d. ruruima* envenomings, 13.5% of the patients presented acute renal failure (serum creatinine >1.5 mg/dL) (26). The frequency of acute renal failure was 18% in Minas Gerais (124) and 12.9% in São Paulo (118). In Central Brazil, 29% of the patients developed acute renal failure; of those, 24% required dialysis, and 10% died (125). In São Paulo, 20% of children envenoming developed acute tubular necrosis, and half of them needed hemodialysis (117). Delay for antivenom treatment (124, 125) and CK at admission >2000 U/L (125) were risk factors for acute renal failure development. The effect of age is controversial (124, 125). In a SINAN-based cross-country comparison, the frequency of acute renal failure was significantly higher in *C. d. ruruima* envenomings than in *C. d. cascavella*, *C. d. terrificus*, and *C. d. collilineatus*, even after adjustment by age, sex, and time to medical care (**Figure 4**).

Experimental models using *C. durissus* subspecies’ venoms and isolated toxins showed important renal alterations, for which several mechanisms are proposed (129–132). Possibly the most critical concern is the damage to skeletal muscle by the myotoxins crotoxin and crotamine, as discussed above. As the major crotoxin-induced product into the circulation, myoglobin is responsible for inducing renal vasoconstriction, formation of intratubular casts, and the direct toxicity to kidney tubular cells, which can be followed by acute kidney injury (AKI) (133). *C. durissus*-induced inflammation is another possible mechanism associated with kidney function; previous studies have shown that inflammatory mediators released from venom-treated macrophages were responsible for renal disturbances (134, 135). Although no studies have been conducted to associate *Crotalus*-induced renal abnormalities with coagulation disorders, this is an important issue to be considered. As a consequence of intravascular coagulation and hemostatic components consumption observed in *C. durissus* patients (115, 136), vascular ischemia due to microthrombi deposition (118) could be responsible for impaired renal functions as observed in similar cases of *Bothrops* accidents (137).

Among venom components, crotoxin was responsible for significant changes in renal function, followed by serine protease inducing mild alterations and CTL with no effects (129). Therefore, the high frequency of AKI in *C. d. ruruima* patients could be a result of myolisis, coagulation disturbances, and inflammation, also with elevated frequency in patients from Roraima. In contrast, crotoxin as the major venom component can play an important role in these events’ physiopathology.

#### Coagulation Disorders

Coagulopathy in *Crotalus* envenomings is attributed to the presence of hemostatically active components in the venom, which can lead to hypofibrinogenemia and unclottable blood (123). Unclottable blood is observed in most of the *Crotalus* envenomings in Brazil, but systemic bleeding seems to be less common (116, 118, 124, 125, 138). In their case series, Cupo and colleagues did not find hemorrhage or thrombosis in the necroscopic analysis (117). Bucaretchi and colleagues (2013) report a case of juvenile *C. d. terrificus* envenoming that evolved with coagulopathy as the main systemic manifestation, without systemic bleeding (136). Also, local bleeding persisting on admission is not a common feature in *Crotalus* envenomings. In Roraima, however, the proportion of the patients presenting to the hospital with bleeding at the bite site ranged from 12.5% (115) to 24.3% (26). In *C. d. ruruima* envenomings, unclottable blood was reported in 62.5% and hypofibrinogenemia in 50% of the patients (115). One fatal case of rattlesnake envenoming attended in Roraima evolved with increased prothrombin time, severe thrombocytopenia, and macroscopic hematuria (59). In the patients reported to SINAN-Roraima, clotting time was prolonged in 55.8%, and systemic bleeding was reported in 9.1% of the patients (**Table 3**). In the cross-country comparison using the SINAN database, blood unclottability was significantly more prevalent in *C. d. ruruima* compared to other *C. durissus* subspecies. Furthermore, systemic bleeding was more frequent in *C. d. ruruima* compared to *C. d. cascavella*, *C. d. terrificus*, and *C. d. collilineatus* subspecies (**Figure 4**). Statistical analysis revealed that systemic bleeding was associated with unclottable blood in this group of patients (OR=4.77, CI95% 1.28-17.70; P=0.021).

**Table 3:** Clinical characteristics of *C. d. ruruima* envenomings reported in the state of Roraima, northern Brazilian Amazon, from 2010 to 2019.

Variable	Number	%
**Local manifestations**		
Pain (n=364)	348	95.6
Edema (n=363)	297	81.8
Ecchymosis (n=358)	21	5.9
**Systemic manifestations**		
Neuroparalysis (n=376)	83	22.1
Myolysis (n=378)	61	16.1
Bleeding (n=375)	34	9.1
Vomiting/diarrhea (n=374)	31	8.3
**Clotting time (n=113)**		
Normal	50	44.2
Prolonged	63	55.8
Severity grade on admission* (n=381; 96.2%)		
Mild	151	39.6
Moderate	170	44.6
Severe	60	15.7
**Antivenom administration (n=381; 96.2%)**		
Underdosage	155	40.7
As recommended	226	59.3
**Local complications**		
Secondary infection (n=345)	11	3.2
Necrosis (n=343)	4	1.2
Compartment syndrome (n=344)	2	0.6
Functional loss (n=343)	3	0.9
Amputation (n=343)	1	0.3
**Systemic complications**		
Acute renal failure (n=336)	33	9.8
Respiratory failure (n=335)	8	2.4
Sepsis (n=335)	2	0.6
Shock (n=334)	5	1.5
**Death (n=350)**		
Yes	4	1.1

*According to Brazilian Ministry of Health guideline (139).

Clotting time, usually performed as the Lee-White clotting time (LWCT) or the 20-minute whole-blood clotting test (20WBCT) usually employed to determine the clotting time, are used worldwide as reliable, low- cost techniques to assess snakebite coagulopathy. Prolonged clotting time assesses the consumption/deficiency in coagulations factors, as well as alterations in platelet count and function (140). The venom is a cocktail of toxins capable of interefering with hemostatic components, leading to coagulopathy. Prolonged clotting times are associated with antagonistic behavior of toxins, which can induce anticoagulant activity, as described for crotoxin and crotamine (79, 141), and procoagulant toxins, reported by serine proteases with thrombin-like activity, which induces the consumption of coagulation factor and hypofibrinogenemia (78, 79). The overall activity induces blood incoagulability, an important factor in bleeding genesis. Another relevant element in hemorrhagic events consists of platelet depletion and the impaired function, majorly promoted by the platelet aggregation agonist convulxin (85), with the backup of others toxins such as crotoxin and crotamine (97, 142) working as agonist and antagonist of platelet aggregation, respectively.

Another interesting aspect concerns the possible role of SVMP from *C. durissus* subspecies venom acting as hemorrhagins. Although their biological activities have not been investigated, SVMP found in North-America in *Crotalus* venoms have been described as inducing local hemorrhage through vessel basement membrane degradation (93), as also observed in *Bothrops* species (143). Considering its abundance in *C. d. ruruima* compared to other venoms, this group of toxins could be associated with the high frequency of bleeding events in patients from Roraima state.

#### Neurological Disorders

Neurological impairment observed in *C. durissus* envenomings is associated with the action of neurotoxins, which are found to act in both peripheral and central nervous systems (96, 144, 145). In the clinical description from the Extra-Amazonian region, neuroparalytic signs, especially the neurotoxic facies/palpebral ptosis, are the most frequent signs of *Crotalus* envenomings. In general, palpebral ptosis is observed in ~70% of the patients (118, 124) and in ~90% of the children (116, 117). Ophthalmoplegia and diplopia are also very frequent, appearing in 95% of general patients and 81% of the children, respectively (117). In the cases reported in the state of Pará, these neurological manifestations are present in almost all patients (126, 146, 147). Bucaretchi and colleagues report an envenoming case caused by a juvenile *C. d. terrificus* that evolved with coagulopathy as the main systemic manifestation, without neuromyotoxic features normally associated with bites by adult specimens (136). In *C. d. ruruima* envenomings, the frequency of palpebral ptosis ranged from 24.3 (26) to 50% (115), and diplopia was seen in 21.6% of the patients (26). Dizziness, difficulty in walking, and muscle weakness are manifestations less frequent in *Crotalus* snakebites, including those caused by *C. d. ruruima* (26, 59, 115). In the patients reported to SINAN-Roraima, neuroparalytic signs were detected in 22.1% of the patients (**Table 3**). Compared to other *Crotalus* subspecies, neuroparalytic signs were significantly less prevalent in *C. d. ruruima* compared to other *C. durissus* subspecies, except when comparing to *C. d. durissus* (**Figure 4**).

Respiratory failure is also a rare complication from *C. durissus* snakebites, being associated with severe cases of envenomings resulting from paralysis of the rib cage and diaphragm muscles (148, 149). A previous report on two cases of *C. d. terrificus* snakebite showed that respiratory manifestations emerged within the first 48 hours and were characterized by dyspnea, tachypnea, use of accessory muscles of respiration, and decreased blood pH and imbalanced pO_2_ pCO_2_ levels (148). SINAN database analysis revealed that the frequency of this complication ranged from 0% in *C. d. marajoensis* to 2.4% in *C. d. ruruima* envenomings. The frequency of respiratory failure was significantly higher in *C. d. ruruima* than in *C. d. terrificus* envenomings (0.5%) (**Figure 4**).

As neurological manifestations and respiratory failure are both associated with the venom neuroxicity, in which crotoxin plays a major role (127), it is possible that the difference in neurotoxic activity could be associated with the singular potency of crotoxin found in each venom. Cavalcante and colleagues showed that crotoxin isolated from *C. d. cumanensis* (inhabit Colombia, Venezuela, and Caribbean coast) presents a more potent neuromuscular blockade effect on mice phrenic-diaphragm preparations when compared to that of *C. d. ruruima* (68).

#### Other Manifestations

Gastrointestinal manifestations, such as nausea (21.6%), vomiting (8.1 to 12.5%), and abdominal pain (8.1%), are reported in *C. d. ruruima* envenomings (26, 115). Necrosis, compartment syndrome, functional loss, and amputation were rare, and their frequencies were similar between envenomings caused by different *C. durissus* subspecies. Headache prevalence ranged from 12.5 (115) to 24.3% (26); dizziness in 12.5% (115); and fever in 8.1% of the patients (26).

#### Risk Factors for Severity

In Brazil, severity classification of *Crotalus* envenomings takes into account the neurological and myolytical signs and symptoms, added to the presence of acute renal failure (139) (**Table 4**). However, bite site manifestations and coagulopathy are not listed as predictors of severity in the Brazilian guideline.

**Table 4:** Severity classification of *Crotalus* bites according the Brazilian Ministry of Health.

Signs/symptoms	Case severity		
	Mild	Moderate	Severe
**Neuroparalytic manifestations (myasthenic facies and others)**	Mild	Evident	Evident
**Myalgia**	Absent	Mild	Evident
**Dark urine**	Absent	Mild	Evident
**Oliguria/anuria**	Absent	Absent	Evident

Source: Brazilian Ministry of Health (139).

**Table 5** summarizes the results of the univariable and multivariable logistic regression models evaluating factors associated with severity in *C. d. ruruima* envenomings reported to SINAN-Roraima from 2010 to 2019. Antivenom underdosage [RR=1.91 (95%CI=1.18–3.09); (p=0.008)], time to medical care >6 hours [RR=1.85 (95%CI=1.14–2.99); (p=0.013)], and Amerindian ethnicity [RR=1.84 (95%CI=1.04–3.26); (p=0.036)] were independently associated with the risk of severity. Bites in lower limbs [RR=0.44 (95%CI=0.26–0.76); (p=0.003)] were associated with protection from severity.

**Table 5:** Factors associated with severity in *C. d. ruruima* envenomings reported in the state of Roraima, northern Brazilian Amazon, from 2010 to 2019.

Severity#	RR	95% CI	*P*	aRR	95%CI	*P*
Underdosage	1.69	1.06-2.69	**0.026**	1.91	1.18-3.09	**0.008**
Time to care >6 hours	1.79	1.11-2.91	**0.018**	1.85	1.14-2.99	**0.013**
Amerindian ethnicity	1.45	0.85-2.48	**0.166**	1.84	1.04-3.26	**0.036**
Site of the bite (lower limbs)	0.51	0.30-0.87	**0.014**	0.44	0.26-0.76	**0.003**
Schooling (years)						
Illiterate	1					
≤4	0.81	0.30-2.19	0.687			
5-8	1.03	0.37-2.87	0.950			
>8	0.94	0.34-2.57	0.913			
Gender (Women)	0.67	0.35-1.27	0.227			
Work-related	1.35	0.69-2.66	0.371			
Rural zone	1.37	0.46-4.07	0.568			
Age (years)						
≤15	1					
16-45	0.97	0.55-1.67	0.906			
46-60	1.08	0.50-2.33	0.842			
>60	1.09	0.44-2.72	0.844			

Proportions of severe cases and deaths were compared by Chi-square test (corrected by Fisher’s test if necessary); differences were considered statistically significant for p<0.05. The crude Relative Risk (RR) with its respective 95% Confidence Interval (95% CI) was determined considering severity and death as the dependent variables. Logistic regression was used for the multivariable analyses, and the adjusted RR with 95% CI were also calculated. All variables associated with the outcomes at a significance level of p<0.20 in the univariable analysis were included in the multivariable analysis. Statistical significance was considered if p<0.05 in the Hosmer-Lemeshow goodness-of-fit test. aRR, Adjusted Relative Risk. Bolded values represent variables significantly associated to severity.

Antivenom underdosage was previously reported as a risk factor for death in the Brazilian Amazon (150), being more frequent in Amerindian patients (114). Incomplete treatments may be associated to with the lack of trained health professionals assisting snakebites and antivenom shortage, forcing professionals to rationalize the limited stock among patients. As also discussed in previous reports (150, 151), late medical care was found to be a risk factor of severity. In these cases, venom toxins acting longer in the body increase the possibility of systemic complications due to more intense myolysis (59, 117). Amerindian ethnicity was also independently associated with the risk of severity, it is necessary to consider the main challenges with cultural, social, and economic disparities between Amerindians and non-Amerindians to understand this association. Firstly, Amerindian villages have their own and very verticalized health system, presenting a low performance in treating snakebites resulting in a higher frequency of long-term disabilities and deaths in these groups (114). Moreover, traditional therapeutic practices within the community involving healers such as shamans, those with knowledge of medicinal plants, and animal-based medicines are often provided in the villages (152). This has a consequence in the time to medical assistance, and the use of deleterious procedures is not discarded (114). Bites in lower limbs were independently associated with milder envenomings. We do not understand the mechanism of this finding, but it may be related to a different bioavailability profile of the venom in the patient’s body according to the bite site. In other studies, older age was associated with severity in rattlesnake envenomings (113, 153). This association was not found in *C. d. ruruima* envenomings reported to SINAN-Roraima, possibly because the number of cases in patients ≥61 years was very small. Indeed, statistical analysis revealed that cases in the state of Roraima occurred mostly in the younger population, as compared to other regions of Brazil (P<0.0001).

## Therapeutics Against *C. d. Ruruima* Envenomings

### Specific Treatment

Specific antivenom is crucial for the efficiency of the treatment of rattlesnake envenomings (154). For the treatment of *Crotalus* envenomings in areas where *C. d. ruruima* occurs, there are three types of antivenoms, two produced in Brazil and one in Venezuela. Characteristics of these products are described in **Table 6**. Although the Brazilian antivenoms are produced using only one or two rattlesnake subspecies venoms (*C. d. terrificus* 100% or *C. d. terrificus* 50% plus *C. d. collilineatus* 50%), both have been used to treat envenomings caused by all the six *Crotalu*s subspecies in Brazil (*C. d. durissus*, *C. d. terriﬁcus*, *C. d. cascavella*, *C. d. ruruima*, *C. d. marajoensis*, and *C. d. collilineatus*) (26, 149, 155). The three producers of crotalid antivenoms in the country follow the guidelines of the *Agência Nacional de Vigilância Sanitária* (Brazilian National Health Surveillance Agency, ANVISA). While each producer uses its own crotalid venoms, all of them use crotamine-abundant venoms in their antigen mixtures (156) (for details, see Section 2). The antivenoms are distributed by the government free of charge. The dosage can vary according to the envenoming severity, though there are standardized recommendations of dosage according to the severity of the case (157). Likewise, Venezuelan antivenom is produced using *C. d. cumanensis* venom, and it is used to treat *Crotalus* envenomings caused by different subspecies occurring in Venezuela (158). Although with adequate, specific and general treatment, most victims envenomed by rattlesnakes can recover and survive, there is some evidence suggesting that the *Crotalus* antivenom cannot neutralize all the deleterious actions of the venom (49). Although crotalid antivenom was effective in neutralizing the lethal, myotoxic, and *in vitro* coagulant activities of the venoms of *C. d. terrificus*, *C. simus*, and *C. d. cumanensis* (159), as well as the lethal effect of *C. d. ruruima* (49) and *C. basiliscus* venoms (160), this antivenom was ineffective in the neutralization of hemorrhagic activity of *C. d. ruruima* venom (49, 159). Hemorrhage was inhibited in the experimental *C. d. ruruima* envenoming only by using the *Bothrops-Crotalus* antivenom, possibly due to the sharing of hemorrhagic toxins with *Bothrops* species (49). Follow-up of four patients envenomed by *C. d. ruruima* has shown that neurotoxic signs ceased, and hemostasis parameters and CK values returned to normal 24 hours after treatment with the *Bothrops-Crotalus* antivenom (115). Otherwise, preliminary reports from the Snakebite Roraima group (Boa Vista, RR, Brazil— www.snakebiteroraima.com) have documented that, in some *C. d. ruruima* envenoming cases, the antivenom does not seem to reverse the venom-induced signs (59). Indeed, antivenomic studies or detailed *in vivo* neutralizing assays with this subspecies venom to elucidate the antivenom efficacy are lacking. Venomics results (see **Table 1**) suggest that different subspecies venoms induce a different proportion of specific antibodies when inoculated in horses; thus, the antivenom manufactured for *C. d. terrificus* and *C. d. colillineatus* venoms may not contain neutralizing antibodies for all *C. d. ruruima* toxins.

**Table 6:** *Crotalus* antivenoms available in areas where *Crotalus durissus ruruima* area reported, in Brazil and Venezuela.

Laboratory (Country)	Antivenom	Description of the antivenom	Venoms used for production	Dosage
Butantan Institute (IBU), Vital Brazil Institute (IVB), and Ezequiel Dias Foundation (FUNED) (Brazil)	*Crotalus* AV	Each vial contains heterologous horse F(ab’)2, neutralizing at 15 mg of the reference venom of *C. d. terrificus*, in mice, phenol (35 mg maximum) and physiological solution 0.85% q.s. 10 mL	*C. d. terrificus* 50.0% plus *C. d. collilineatus* 50.0% (IBU) or *C. d. terrificus* 100% (IVB and FUNED)	5 vials to mild cases, 10 vials to moderate cases, 20 vials to severe cases
Butantan Institute (IBU), Vital Brazil Institute (IVB), and Ezequiel Dias Foundation (FUNED) (Brazil)	*Bothrops-Crotalus* AV	Each vial contains heterologous horse F(ab’)2, neutralizing at least 50 mg and 15 mg of the reference venoms of *Bothrops jararaca* and *C. d. terrificus*, respectively, in mice, phenol (35 mg maximum) and physiological solution 0.85% q.s. 10 mL	*Bothrops* genus (*B. jararaca* 50.0%, *B. alternatus* 12.5%, *B. jararacuçu* 12.5%, *B. moojeni* 12.5% and *B. neuweidi* 12.5%), *Crotalus* genus (*C. d. terrificus* 50.0% plus *C. d. collilineatus* 50.0% - IBU, or *C. d. terrificus* 100% - IVB and FUNED)	5 vials to mild cases, 10 vials to moderate cases, 20 vials to severe cases
BIOTECFAR - Biotechnology Center, Venezuela Faculty of Pharmacy of the Venezuela University Center (Venezuela)	*Bothrops-Crotalus* AV	Each vial contains heterologous horse F(ab’)2, neutralizing at least 20 mg of the reference venom of *Bothrops colombiensis* and 15 mg of the reference venom of *C. cumanensis*, in mice, phenol (30 mg maximum) and physiological solution 0.85% q.s. 10 mL	*Bothrops colombiensis* and *C. d. cumanensis*	10 vials to moderate cases and 15 vials to severe cases

The need for a cold chain and physicians to prescribe antivenoms usually restricts access to effective snakebite treatment (161). However, as presented above, *C. d. ruruima* envenomings are reported mostly in Amerindian populations, and it is necessary to consider challenges related to cultural, social, and economic disparities between Western and indigenous cultures, contributing to late medical care among the latter (114). In Roraima, some Yanomami sanitary districts have antivenom available for mild to moderate snakebite cases (162), but in the other indigenous groups, referral to urban areas is needed. The transfer of the indigenous to urban areas is a critical event in these individuals lives, due to the breakdown of relations with the village and eating habits, causing treatment refusals (114). From an economic point of view, the public health system spends thousands of dollars per year to transport indigenous patients by plane or helicopter (~US$600 and ~US$900/hour of flight, respectively), in addition to hospital costs and loss of productivity, which are also very high (US$8 million in 2015, in the Amazon region) (163). Besides the specific treatment, supportive assistance during severe rattlesnake treatment is crucial but possible only in the capital, Boa Vista. Strategies that guarantee this affected population’s cases timely access to treatment and proper training of health professionals who deal with indigenous populations are key to providing the best care to the indigenous communities. In a previous study, we discuss a plan for the decentralization of antivenom treatment to local healthcare facilities as an intervention to increase the indigenous population’s access to proper healthcare (114).

An important fact to be mentioned is the absence of antivenoms in the health facilities of neighboring countries where *C. d. ruruima* envenomings are also common. In Guyana, for example, health authorities have only recently announced the acquisition of antivenoms (the polyvalent antivenom PoliVal-ICP, Instituto Clodomiro Picado, Costa Rica), which are available only in Georgetown, the country’s capital (164). The country has one of the highest case fatality rates (~ 8%) on the continent, which is explained by the fact that few patients visit the health facilities offering proper treatment (165). Envenomings by *C. d. ruruima* are reported in the Guianan savanna, especially in the Lethem region, on the border with Brazil (59). In the absence of antivenoms in these localities, and because of the nearly nonexistent evacuation possibilities to the capital, these patients seek treatment in Brazil. In Venezuela’s case, the Laboratorios BIOTECFAR, a public producer maintained by Universidad Central de Venezuela, responsible for the production of antivenoms in the country, has experienced difficulty maintaining continuous production in the last years (158). The shortage of antivenom in Venezuelan is suggested as the cause of patients seeking treatment in Brazil after severe snakebites (166).

### Heterologous Antivenom Disadvantages and Next-Generation Crotalid Antivenoms

Although promising results suggest that the PLA_2_ inhibitors varespladib and methyl-varespladib are effective in preventing neurotoxic manifestations induced by *C. d. terrificus* (167), crotalid heterologous antivenoms are the only available effective treatment for rattlesnake envenomings in endemic areas. As other heterologous antivenoms, they may present undesirable problems, such as anaphylactic reactions, and serum sickness. Moreover, many of the antibodies from antivenoms (~70%) are composed of non-neutralizing antibodies [for review, see (168)]. Thus, it is evident that the century-old crotalid therapy introduced by Vital Brazil in 1901 (169) needs to be improved. The information on the safety of *Crotalus* antivenoms is scarce, but it was estimated that 80% of patients developed developed an early, adverse event of early adverse event after using the available Brazilian *Crotalus* antivenom, with no significant difference in the frequency of patients with early reactions between the groups that were and were not pretreated with antihistamines and corticosteroids (116). Even though the antivenom purification process has, over time, contributed to reducing this frequency to around 20% today (115), the possibility of early reactions generates fear among health professionals and is a major obstacle for antivenom decentralization to remote areas (114).

The first approach to decreasing the size of the immunocomplexes generated by heterologous antivenoms and preventing side effects (e.g., serum sickness) treated all immunoglobulins (IgGs) with specific enzymes. This methodology results in antigen-binding fragments F(ab’)_2_s or Fabs (170), which are much smaller, corresponding to 115 and 50 kDa, respectively (a whole IgG presents ~150 kDa) (171). At the moment, most of the animal-derived crotalid antivenoms are composed of antibody fragments (e.g., CroFab (172), BIOTECFAR from Venezuela, and both crotalid antivenoms from Brazilian producers). Although the antibody size reduction was important for improving antivenom therapy (173), the molecules continue to cause side effects in keeping their heterologous nature. Indeed, the Instituto Clodomiro Picado from Costa Rica still produces crotalid antivenoms composed of whole IgGs (174).

Animal plasma-derived antivenoms will continue to be the cornerstone of envenoming therapy for many years to come, and it may indeed be warranted to further improve these life-saving medicines e.g. *via* improved downstream processing or improved immunization approaches (175). However, further into the future, it is not unlikely that recombinant DNA technology and the application of human monoclonal antibodies and/or nanobodies may find their way into the field (176). Indeed, the development of monoclonal antibodies to treat envenomings caused by venomous animals is now being extensively investigated (177–181), although none have so far been tested in human patients. Here, we will explore only the discoveries regarding experimental crotalid antivenoms.

Antibody phage display (182) has been used as the main successful technology to generate monoclonal antibodies against toxins. In 1995, the technique was first used for the discovery of monoclonal antibody fragments against animal toxins (176). Interestingly, the target toxin was a crotoxin obtained from the snake *C. d. terrificus* (183). Although the mouse-derived library construction and the antibody panning were performed appropriately, the selected antibody failed to inhibit the PLA_2_ activity induced by crotoxin. In 1997, the first human monoclonal antibody generated by phage display against crotoxin was obtained. The research group discovered scFvs targeting crotoxin from a human semi-synthetic antibody phage display library, and the antibodies demonstrated to bind the toxin in ELISA assays (184).

In 2009, a non-immune human single-chain fragment variable library (Griffin.1) was used to select antibodies against *C. d. terrificus* PLA_2_s. Two clones demonstrated the ability to partially inhibit the PLA_2_ activity *in vitro*, to reduce the myotoxic and edema activities of basic crotoxin *in vivo*, and to inhibit the lethality of *C. d. terrificus* venom in an experimental envenoming (185). Later (2018), using previous phage display selections, the same research group discovered scFv clones able to cross-inhibit *in vitro* indirect hemolysis and plasma-clotting effects of *C. d. terrificus* and *Bothrops jararacussu* venoms (186). Using a phage display peptide library, Titus and colleagues (2017) also developed peptides against a PLA_2_ consensus peptide from North American rattlesnakes, *C. adamanteus*, *C. atrox*, *C. scutulatus*, *Agkistrodon piscivorus*, and *A. contortrix laticinctus*. PLA_2_s from these venoms had their *in vitro* enzyme activity slightly reduced by the selected peptides by 40% or less (187).

Thus far, the discovery of monoclonal antibodies and peptides have only yielded inhibitors of crotoxin and PLA_2_s from *Crotalus* spp, with no reports on monoclonal antibodies against crotamine or other rattlesnake venom compound. Part of the explanation for this may be that PLA_2_s seem to dominate as antigens compared to other venom components. Campos et al. demonstrated that when snake whole venoms were used as targets in phage display panning rounds, antibodies were primarily selected against PLA_2_ proteins (180). To overcome this difficulty, key toxins that are important to neutralize, but which are not dominant in neither phage display campaigns or immunization schemes, can be carefully isolated or recombinantly expressed to provide more control over the antibody selection or immunization process (188–190). In the future, such efforts could hypothetically both be used to identify antibodies that could be used to fortify existing crotalid antivenoms (191), and thereby extend their species coverage, or improve their efficacy against non-immunogenic components (190). Finally, it is speculated that efforts focusing on the discovery and development of monoclonal antibodies against *Crotalus* toxins could possibly enable the design of fully recombinant antivenoms based on oligoclonal mixtures of antibodies targeting all medically important venom toxins (192). If such efforts became fruitful, they could lead to better envenoming therapies with improved safety and efficacy, allowing for the deployment of such products in remote areas due to a decreased risk of anaphylactic shock and other serious immunological reactions (193). Such potential improvements could have a profound impact on rural communities in regions such as the Amazon, however, it is important to stress that all research efforts reported so far are in very early development. If monoclonal antibodies will one day find their way into clinical development, this will likely be several years into the future. Meanwhile, plasma-derived antivenoms will remain the mainstay of envenoming therapy against *Crotalus* spp.

## Concluding Remarks

*C. d. ruruima* bites represent an important public health issue in the northernmost state of Brazil, Roraima, given the predominance of this snake subspecies in the region and the severity of its bite. Envenoming pathophysiology includes pain, edema, myolysis, bleeding, uncoagulable blood, renal dysfunction, and respiratory impairment, which have been observed more frequently in patients envenomed by *C. d. ruruima* compared to other *C. durissus* subspecies. We identified that antivenom underdosage, time to medical care, and being part of an indigenous population are risk factors of rattlesnake envenoming in Roraima state. Therefore, *C. d. ruruima* snakebites deserve special attention in the clinical and therapeutic management by health professionals. Also, better strategies for accessing specific antivenom through public distribution policies are needed. It is evident that *C. d. ruruima* venom is still among the least explored venoms from *Crotalus* species, with little existing data on its toxins and induced effects.

## Author Contributions

MP, PB, and WM conceived the main idea of this work. AR, PV, RF, IO, IF, and ES conducted the bibliography search. MP, PB, IO, MS, and WM designed and wrote most of this review’s topics. VS performed the statistical analysis. FC designed the figures of this review article. JS, FC, AL, and FW corrected the manuscript and provided important contributions during the development of this work. All authors contributed to the article and approved the submitted version.

## Funding

We thank *Fundação de Amparo à Pesquisa do Estado de São Paulo* (FAPESP, São Paulo Research Foundation; scholarships to IO no. 2017/03580-9 and FC no. 2017/14035-1), *Conselho Nacional de Desenvolvimento Científico e Tecnológico* (CNPq, The National Council for Scientific and Technological Development, scholarship to MP no. 307184/2020-0 and WM n. 309207/2020-7) and the *Coordenação de Aperfeiçoamento de Pessoal de Nível Superior—Brasil* (CAPES, Finance Code 001, scholarship to IF). WM acknowledges funding support from *Fundação de Amparo à Pesquisa do Estado do Amazonas* (PAPAC 005/2019, PRÓ-ESTADO and Posgrad calls). MP (Snakebite Roraima project coordinator) acknowledges funding support from Hamish Ogston Foundation—Global Snakebite Initiative.

## Conflict of Interest

The authors declare that the research was conducted in the absence of any commercial or financial relationships that could be construed as a potential conflict of interest.
